# Supplementary material for: Modeling glioblastoma heterogeneity as a dynamic network of cell states
Source: Mol Syst Biol. 2021 Sep 16;17(9):e10105. doi: 10.15252/msb.202010105 (PMC8444284; doi:10.15252/msb.202010105)
Supplement: Supplementary file 6 — Source Data for Figure 5 [file MSB-17-e10105-s004.zip › Figure5A_sourcedata/GSEA_3017/nosigdb_stateE.GseaPreranked.1622795031093/GANDAL_AUTISM_UP_PMID29439242.html]

Details for gene set GANDAL\_AUTISM\_UP\_PMID29439242[GSEA]

|  || Dataset | state63017 |
| Phenotype | NoPhenotypeAvailable |
| Upregulated in class | na\_pos |
| GeneSet | GANDAL\_AUTISM\_UP\_PMID29439242 |
| Enrichment Score (ES) | 0.6744722 |
| Normalized Enrichment Score (NES) | 3.3490498 |
| Nominal p-value | 0.0 |
| FDR q-value | 0.0 |
| FWER p-Value | 0.0 |
Table: GSEA Results Summary

  

Fig 1: Enrichment plot: GANDAL\_AUTISM\_UP\_PMID29439242      
 Profile of the Running ES Score & Positions of GeneSet Members on the Rank Ordered List

  

| PROBE | GENE SYMBOL | GENE\_TITLE | RANK IN GENE LIST | RANK METRIC SCORE | RUNNING ES | CORE ENRICHMENT || 1 | CYR61 |  |  | 3 | 1.169 | 0.0751 | Yes |
| 2 | IGFBP5 |  |  | 4 | 1.015 | 0.1430 | Yes |
| 3 | CCL2 |  |  | 5 | 0.975 | 0.2083 | Yes |
| 4 | ANXA1 |  |  | 12 | 0.826 | 0.2572 | Yes |
| 5 | CD44 |  |  | 14 | 0.806 | 0.3101 | Yes |
| 6 | CEBPD |  |  | 15 | 0.791 | 0.3631 | Yes |
| 7 | CRYAB |  |  | 16 | 0.788 | 0.4159 | Yes |
| 8 | ANXA2 |  |  | 18 | 0.733 | 0.4639 | Yes |
| 9 | IFITM3 |  |  | 26 | 0.682 | 0.5020 | Yes |
| 10 | GLIS3 |  |  | 31 | 0.630 | 0.5398 | Yes |
| 11 | TAGLN2 |  |  | 32 | 0.621 | 0.5815 | Yes |
| 12 | EMP1 |  |  | 40 | 0.584 | 0.6130 | Yes |
| 13 | IFITM2 |  |  | 46 | 0.536 | 0.6435 | Yes |
| 14 | S100A10 |  |  | 51 | 0.527 | 0.6745 | Yes |
| 15 | PTTG1IP |  |  | 225 | 0.280 | 0.5058 | No |
| 16 | SPP1 |  |  | 261 | 0.257 | 0.4852 | No |
| 17 | ZFP36L1 |  |  | 287 | -0.255 | 0.4752 | No |
| 18 | TXNIP |  |  | 404 | -0.284 | 0.3685 | No |
| 19 | SLCO4A1 |  |  | 478 | -0.308 | 0.3100 | No |
| 20 | KIF20A |  |  | 523 | -0.321 | 0.2838 | No |
| 21 | FKBP5 |  |  | 536 | -0.328 | 0.2928 | No |
| 22 | MTHFD2 |  |  | 625 | -0.372 | 0.2224 | No |
| 23 | TM4SF1 |  |  | 823 | -0.619 | 0.0504 | No |
| 24 | HIST1H1C |  |  | 936 | -1.220 | 0.0108 | No |
Table: GSEA details [plain text format]

  

Fig 2: GANDAL\_AUTISM\_UP\_PMID29439242: Random ES distribution      
 Gene set null distribution of ES for **GANDAL\_AUTISM\_UP\_PMID29439242**
